# Supplementary material for: Are carbon emissions trading and green financial instruments synergistic? -Comprehensive quantitative research based on content analysis
Source: PLoS One. 2024 Mar 7;19(3):e0298601. doi: 10.1371/journal.pone.0298601 (PMC10919601; doi:10.1371/journal.pone.0298601)
Supplement: S1 Data — (ZIP) [file pone.0298601.s001.zip › Data/地级市Z_F工作报告.zip!/╦─┤¿/├α╤⌠╩╨/└┤╫╘╣1⁄2╓┌║┼í╛┬φ┐╦╩2╛▌═°í┐_2018.docx]

各位代表：

现在，我代表市人民政府，向大会报告工作，请予审议，并请市政协委员提出意见。

2017年工作回顾

过去一年，在党中央国务院、省委省政府和市委坚强领导下，在市人大、市政协监督支持下，我们*学习贯彻习近平新时代中国特色社会主义思想和党的十九大精神，牢牢把握稳中求进工作总基调，坚持科学发展、加快发展工作取向，统筹抓好稳增长、促改革、调结构、惠民生、防风险各项工作，圆满完成市七届人大二次会议确定的目标任务，经济社会发展取得显著成效。

——经济实力跃上新台阶。实现地区生产总值2074.75亿元、增长9.1%，成为全省首个经济总量突破2000亿的地级市，在西部同类城市中的排名由第11位上升至第8位；增速居全省第1位，是自1999年以来在全省最高排位。规上工业增加值增长10.5%，居全省第4位，是自2004年以来在全省最高排位。服务业增加值增长10.8%，是自建市以来首次排名全省第1位。

——科技城建设积极推进。科技城“十三五”发展规划获得国务院批复。全国首批推广的13条全创经验绵阳贡献2条，全省首批推广的21条经验中涉及绵阳11条。科技创新综合水平指数达到70.64%，科技对经济增长贡献率达到58%。中国工程物理研究院某重大装置等一批国家重大科技专项落地绵阳、加快实施，绵阳国防军工重镇地位进一步巩固。

——改革开放迈出坚实步伐。重点领域和关键环节改革*推进，280项年度改革任务基本完成，10多项改革经验做法得到中央领导和国家部委肯定。成功举办第五届科博会、中国（四川）电商发展峰会。招商引资保持强劲势头，经省上认定的国内省外到位资金431亿元，继续稳居全省第2位。

——人民生活水平不断提升。脱贫攻坚取得决定性进展，北川贫困县摘帽通过省级验收，全市又有176个贫困村退出、31866名贫困人口脱贫，贫困发生率下降到1.14%。城乡居民人均可支配收入分别达到31822元、14752元，增长8.2%、9.2%。城镇新增就业5.72万人，城镇登记失业率控制在3.7%。城乡居民基本养老保险覆盖率达到99.74%。完成城镇危旧房棚户区改造4354套、农村危房改造5263户。

——生态环境状况总体改善。*实施污染防治“三大战役”并取得明显成效。单位地区生产总值能耗下降5.6%。清洁能源消费比重同比提高1.25个百分点。城区空气质量优良天数达到295天，达标率81.7%、同比提高3.5个百分点。森林覆盖率达到53.86%、同比提高0.26个百分点，绿色发展呈现可喜局面。

一年来，我们主要做了以下几个方面工作。

一、坚持把经济建设作为兴市之要，着力稳增长、调结构、提质量，经济发展取得新成效

面对复杂多变的宏观经济形势和激烈的区域竞争态势，我们谋早抓实、精准发力，*推进供给侧结构性改革，大力振兴实体经济，在做大经济总量中优化结构、提升质量，全市经济保持了稳中有进、稳中向好的良好态势。

大力推动三次产业协调发展。把产业作为经济发展的核心，坚持优先发展先进制造业、加快发展现代服务业、大力发展现代农业，严格落实重点产业及空间布局方案，三次产业结构优化调整为14.1:40.4:45.5，经济增长从主要依靠工业拉动转变为工业和服务业共同拉动。*实施工业大企业大集团培育“领航计划”，新增50亿元以上企业2户、10亿元以上企业7户、4亿元以上企业17户。加大中小企业培育力度，新增规上工业企业108户。制定出台改进服务工业企业九条措施，帮助解决企业生产经营中的困难问题，攀长钢结束二十年亏损、实现扭亏为盈，长虹控股公司蝉联川企百强榜首。出台特色园区推进工作方案，促进产业集群发展，全市产业园区规上工业总产值增长17%。坚持加快发展生产**业与提升发展生活**业并重，推动服务业上档升级。服务业对经济增长贡献率达到50.1%，拉动经济增长4.6个百分点。坚持以提高农业供给质量为主攻方向，促进农业提质增效。实现第一产业增加值291.66亿元、增长4%。建成现代特色效益农业标准化基地12.1万亩，现代农业产业园区31个，申报“三品一标”农产品243个，新型农业经营主体达1.7万个。

着力提升发展质效。坚持以高新技术产业和战略性新兴产业为主攻方向，突出抓好电子信息、汽车产业发展，支持新材料、节能环保、高端装备制造等优势产业提升能级，“两新”产业产值占规上工业总产值比重分别达到54.1%、37.5%，同比分别提高0.6和1.5个百分点，继续保持全省领跑优势。全市规上工业企业主营业务收入2723.2亿元、增长18.2%，盈亏相抵后利润总额133.3亿元、增长19.9%。单位工业增加值能耗下降6.7%。在推进“营改增”等减税降费政策的情况下，地方一般公共预算收入达到110.59亿元，同口径增长8.02%，地方一般公共预算收入中税收占比提高1.15个百分点。*开展质量对标行动，加快建设质量强市，主导或参与制修订国家和行业标准10项，新增商标注册1800件。

持续扩大投资消费。发挥投资对优化供给结构的关键性作用。坚持以项目为中心组织经济工作，*推进“项目年”行动，每季度组织全市重大项目集中开工，制定重点项目专员等制度，促进投资持续稳定增长。完成全社会固定资产投资1436.54亿元、增长13.1%，增速全省排名连续四年上升。216个省市重点项目完成投资350.6亿元，投资完成率131.5%。在保持投资强度的同时不断优化投资结构，工业投资增长26.6%，技改投资增长26.8%。增强消费对经济发展的基础性作用，推动传统消费提档升级、新兴消费快速兴起。组织市内企业积极参与“惠民购物全川行”“川货全国行”“迎春购物月”等消费促进活动，社会消费品零售总额突破千亿大关，达到1112.48亿元、增长12.5%。最终消费对经济增长贡献率达到52.4%。

二、坚持创新驱动军民融合，着力促改革、扩开放、添活力，科技城建设取得新进展

纵深推进全创试验。坚持以系统推进全面创新改革试验为引领，努力探索军民融合深度发展的“绵阳路径”，顺利完成147项年度改革任务。建立国内首个“电子信息军民融合创新实验室”、全省首家军民两用技术再研发中心等创新平台。成立国内首家军民融合科技支行。推动中国兵器装备集团自动化研究所转制改企和混合所有制改革取得实质进展。持续开展军民融合企业认定，新增军民融合企业85家、总数达到321家，军民融合企业产值1530亿元、占规上工业总产值比重超过50%。

加快建设国家科技城。认真实施科技城“十三五”发展规划，全面落实部际协调小组第13次会议精神，加快建设国家创新驱动发展“试验田”、军民融合创新“排头兵”、西部地区发展“增长极”。把集中发展区作为城市发展和产业发展战略平台，高水平编制集中发展区总体规划、核心区详细规划及城市设计。推动科技城大道河边段等项目竣工投用，5平方公里启动区基本建成；安昌河综合整治、地下综合管廊等项目积极推进，30平方公里核心区主体框架加快形成。京东方柔性面板生产线、中电科磁性材料产业园等重大产业项目加快建设。*推进大众创业、万众创新上水平，累计认定科技型中小企业超过1万家，新增国家、省级企业技术中心、工程技术研究中心、重点实验室10个。*推进国家知识产权示范城市建设，强化知识产权运用和保护，全市专利申请量10889件，专利授权量4749件，万人发明专利拥有量达到8.54件。游仙入选国家知识产权强县工程示范区。

稳步推进重点领域改革。扎实推进“三去一降一补”，实行水泥企业错峰生产，压减水泥产量345.4万吨。努力减轻企业负担，减免各类税费62.82亿元。着力解决企业融资难题，为63户企业办理应急转贷资金4.94亿元，降低融资成本2500万元；发挥高新技术中小企业贷款风险补偿基金作用，推动银行向62户企业发放贷款4.06亿元。*推进商事制度改革，持续优化营商环境，新登记市场主体4.87万户，其中企业1.26万户。深化农业供给侧结构性改革，稳步推进农村集体产权制度改革试点。加快推进国资国企改革，推动长虹授权经营改革，将15项出资人权力授权长虹控股公司，在四川华丰开展全省首批国有控股混合所有制企业员工持股试点。文化教育、医药卫生、投融资等领域改革取得明显成效。

不断提升对外开放水平。坚持以全面开放推动改革深化和高质量发展。把招商引资作为推动发展“第一抓手”，持续开展精准专业化招商，成功引进汉能、华晨、东旭、360等47个10亿元以上重大产业项目。强化成德绵区域合作，积极融入“一带一路”、长江经济带。新增国际友城和国际友好合作城市5个、总数达到25个。南郊机场年旅客吞吐量达到354.3万人次、增长63.1%。实现外贸进出口总额115亿元。加快企业“走出去”步伐，对外投资总量居全省第2位。

三、坚持城乡统筹区域协调，着力打基础、强支撑、增后劲，城乡面貌呈现新变化

加快构建新型城镇体系。大力推进新型城镇化综合试点，坚持规划引领、统筹推进，加快形成以中心城区为龙头、县城为支撑、重点镇和特色镇为基础的现代城镇体系。推动安州区加快融入中心城区。中心城区新增建成区面积10.4平方公里、新增常住人口5.3万人，达到149.5平方公里、138.1万人。海绵城市试点创建工作加快推进。*实施城建攻坚行动，加快推进“重构一环、贯通二环”，加快建设剑门路西段片区排水综合改造工程，如期建成安昌河石桥铺景观闸坝。实施增绿添绿行动，城市绿地率提高0.5个百分点，达到36.7%。6个县（市）县城建成区面积新增2.1平方公里，达到94.2平方公里，产业承载能力和人口吸纳能力进一步增强。加快推进小城镇建设，江油市青莲镇入选第二批全国特色小镇，三台县西平镇、涪城区龙门镇等21个国家重点小城镇和省级试点镇建设取得新进展。制定实施幸福美丽新村建设规划，新建幸福美丽新村466个。出台《推进农民工融入城市（镇）工作实施方案》，全市常住人口城镇化率、户籍人口城镇化率提高1.5、1.3个百分点，分别达到51%、34.1%。

*实施县域振兴计划。大力实施“五大行动”，着力培育充满活力、特色化、专业化的县域经济，不断壮大县域经济实力。设立50亿元的县域经济产业发展投资基金，助推县域产业发展。县域地区生产总值占全市比重达到78.1%、同比提高0.4个百分点。9个县市区在全省类区县排名稳中有升。涪城连续3年入选全国投资潜力百强区。游仙入选全省第二批工业强县示范县。三台、江油获评全省“三农工作先进县”。平武、北川被命名为全省县域经济发展模范县。

大力推进基础设施建设。加速推进交通攻坚行动，西成高铁正式通车，绵西高速路基工程基本完工，九绵高速全线启动建设，广平高速开工建设，全市在建高速公路总里程达301公里，与已通车高速公路总里程相当。实施S205改线等国省干线公路新建改建项目19个、321公里，完成S101等国省干线公路大中修工程7个、155公里。提前完成涪江三桥维修加固。建设县乡道392公里、村道1266公里，群众出行更加便利。农村公路建设获省政府农田水利基本建设考核第1名，安州被认定为首批“四好农村路”省级示范县。新建乡镇生活污水处理设施39个，乡镇场镇生活垃圾收运实现全覆盖。积极推进武引二期灌区等水利工程建设，新增和恢复改善灌溉面积25万亩。加快推进城乡电网改造，升级改造278个村农村电网。不断完善天然气管网,新增用户1.3万户。*实施“宽带绵阳”建设，新建4G基站超过3000个。

四、坚持以人民为中心的发展思想，着力补短板、惠民生、防风险，民生福祉得到新改善

强力推进脱贫攻坚。把脱贫攻坚作为决胜全面小康社会的头等大事，实施全面决战决胜脱贫攻坚46条硬措施，全市投入36.1亿元，实施扶贫专项23个。加大产业扶贫力度，大力发展特色种养殖、农产品加工、乡村旅游、农村电子商务等产业，带动贫困人口人均增收1275元。完成易地扶贫搬迁2263人。支出医疗救助资金3.2亿元。丘区脱贫攻坚示范区建设取得阶段性成效。对口协作帮扶红原、壤塘、布拖、昭觉四个深度贫困县工作走在全省前列。

全面发展民生和社会事业。在财力十分紧张的情况下，投入资金80.56亿元，实施十项民生工程，办好20件民生实事。“义务教育薄弱学校改造计划”覆盖学校新增78所，惠及学生新增4.1万人。积极推进学前教育优质普惠发展、义务教育优质均衡发展。加大社会保障投入力度，发放低保资金6.35亿元；多方筹集资金44.5亿元，集中解决了4.98万名失地农民参保遗留问题。积极提升医疗卫生水平，开工建设市儿童医院，基层医疗卫生机构规范化建设达标率73%。全面取消公立医院药品加成，为患者减轻药费负担2.85亿元。新增养老机构床位2000张。加快发展文化事业，北川地震遗址保护区成为全省第二家国家级海峡两岸交流基地，《卓玛卓玛》等10项文化作品获国家奖励。居民消费价格同比上涨1.9%，物价水平保持总体稳定。实施功能治堵、工程治堵、管理治堵三大措施，完成治堵缓堵项目35个，交通拥堵问题得到初步缓解。积极推进便民服务设施建设，中心城区新建成农贸市场5个，新增公共停车位1324个，新建改建公厕43座。成功蝉联全国文明城市。

持续提高社会治理水平。*推进法治绵阳建设。加强基层基础工作，努力构建自治、法治、德治相结合的治理体系，基层治理水平不断提升。强化安全生产工作，各类生产安全事故起数、死亡人数、受伤人数同比分别下降19.5%、19.4%、20.5%，未发生重特大生产安全事故。持续推进平安绵阳建设，全市有效接警数下降9.3%，刑事立案数下降25.8%，破案数上升14.9%，平安建设群众满意度居全省第1位。食品安全总体状况满意度连续四年保持全省第1位。成功化解汉龙资产处置、波鸿信贷违约等重大金融风险。圆满完成“8·8”九寨沟地震抗震救灾交通保障、游客疏散和医疗救援任务。

切实加强生态文明建设。出台污染防治三大战役实施方案。制定实施《重污染天气应急预案》，优良天数达标率和PM10、PM2.5年均浓度实现“一升双降”。全面推行河长制，创新设立河道警长制，实现市县乡村四级河长体系全覆盖，县级及以上城市集中式饮用水水源地、涪江绵阳段流域断面水质达标率均达到100%。实施土壤污染防治行动计划，土壤环境质量总体稳定。*实施大规模绿化绵州行动十大工程，新增森林面积8.1万亩。扎实推进环保督察问题整改，中央环保督察组交办的信访案件全部按时办结。

国防动员、民族宗教、外事侨务、新闻出版、广播影视、统计调查、保密、档案、气象、水文、海关、邮政、检验检疫、慈善、红十字、残疾人、妇女儿童、老龄、地方志、住房公积金等工作都取得了新的成绩。

一年来，我们切实加强政府自身建设。坚持依法行政，自觉接受人大、政协监督，主动接受人民群众监督。回应人大代表、政协委员关切，办理人大代表建议325件、政协委员提案713件。成功化解31件多年未能解决、事关人民群众切身利益和企业发展的历史遗留问题，政府公信力不断提升。出台《建设服务型政府行动方案》，不断深化“放管服”改革，全面推行相对集中行政许可权改革，取消市级部门行政许可事项23项、市级行政审批项目前置条件276项。在全省率先推行建设项目全程并联审批，项目审批、竣工验收时间进一步缩短。建立实施重点招商项目和重点企业服务绿卡制度。企业和群众办事更加方便。“12345”市长热线进入全国政务热线“第一方阵”。严格落实中央、省、市转变作风规定及实施细则要求，持之以恒纠正“四风”，“三公”经费下降21.1%。加强行政监察和审计监督，狠抓廉洁从政，持续高压反腐，政纪立案229件、政纪处分229人，政风建设取得新成效。

各位代表！过去一年绵阳经济社会发展取得的成绩来之不易。这是习近平新时代中国特色社会主义思想科学指引的结果，是以习近平同志为核心的党中央、省委省政府和市委坚强领导的结果，是市人大、市政协和人大代表、政协委员监督支持的结果，是全市广大干部群众拼搏实干的结果。在此，我代表市人民政府，向全市各族人民，向人大代表、政协委员，向各民主党派、工商联、无党派人士和各人民团体，向驻绵解放军指战员、武警官兵、政法干警和民兵预备役人员，向港澳台同胞、广大侨胞和海内外华人华侨，向关心支持绵阳改革发展的朋友们及各界人士，表示诚挚的感谢并致以崇高的敬意!

安不忘危、兴不忘忧。我们清醒认识到，我市经济社会发展中还存在一些矛盾和问题。主要表现在：发展不平衡不充分的问题还比较突出；城乡居民收入差距较大，农村基础设施比较薄弱；产业结构有待进一步优化；军民融合体制机制障碍尚未有效破解；金融领域、社会领域风险隐患仍然存在；政府债务较重，收支矛盾突出；民生领域还有不少短板，社会保障水平有待提高；部分企业生产经营还面临一些困难，民间投资增速下滑；营商环境还需进一步改进；部分干部服务意识和担当精神不够。对这些问题，我们高度重视，正采取有力举措，切实加以解决。

2018年工作安排

今年政府工作的总体要求是：以习近平新时代中国特色社会主义思想为指导，*学习贯彻习近平总书记来川视察重要讲话和党的十九大、十九届二中、三中全会精神，认真落实中央、省委、市委经济工作会议部署要求，坚持稳中求进工作总基调，*贯彻新发展理念，紧扣社会主要矛盾变化，按照高质量发展的要求，坚持以供给侧结构性改革为主线，统筹推进稳增长、促改革、调结构、惠民生、防风险各项工作，推动质量变革、效率变革、动力变革，在打好防范化解重大风险、精准脱贫、污染防治“三大攻坚战”上取得扎实进展，促进全市经济社会持续健康发展，加快建设国家科技城和西部经济强市。

今年经济社会发展的主要预期目标是：地区生产总值增长8.5%，规上工业增加值增长10.4%，全社会固定资产投资同口径增长13.5%，社会消费品零售总额增长11.5%，地方一般公共预算收入增长9%，城乡居民人均可支配收入分别增长8%、9%，城镇登记失业率控制在4%以内，居民消费价格涨幅控制在3%以内，完成省政府下达的环境保护和节能减排目标任务。

为实现上述目标，我们将重点抓好以下工作。

一、大力推动高质量发展

坚持质量第一、效益优先，努力构建现代产业体系，继续优化产业结构，持续扩大投资消费，实现经济较快增长和质量效益稳步提高互促共进，加快建设西部经济强市。

推动产业转型升级。实施制造业七个单项行动计划，争创“中国制造2025”国家级示范区。加快发展电子信息和汽车两大支柱产业，力争八大重点产业产值占规上工业总产值比重同比提高2个百分点，“两新”产业产值占规上工业总产值比重分别达到55%、38%。制定实施服务业八大重点产业行动方案和年度计划，持续提升生产**业在服务业增加值中的占比，推动10个项目、15个企业、6个品牌进入全省服务业“三百工程”名录。*实施工业大企业大集团培育“领航计划”，新增50亿元以上企业1户、30亿元以上企业1户、10亿元以上企业4户；加大“四上企业”培育力度，新增“四上企业”357户，其中规上工业企业100户。加大企业上市挂牌培育力度，促进企业利用资本市场做大做强。*推进“51025”重点园区培育计划和“园区千百亿工程”，力争园区经济占全市工业经济比重达到82%，园区产业集中度同比提升1个百分点。

促进有效投资。继续深化“项目年”行动，加快推进省市重点项目，形成梯次衔接、压茬推进的良好局面。持续优化投资结构，力争工业投资和技改投资均同口径增长20%以上。落实鼓励民间投资政策措施，新推出一批PPP、TOT、TBT项目，真正让民间资本进得来、能发展，力争民间投资同口径增长10%以上。合理引导市场预期和购房行为，促进房地产市场平稳健康发展。

积极扩大消费。实施改善消费供给专项行动，新增限额以上批零住餐企业100户。扩大信息消费，发展远程定制、体验分享等新模式。加速升级旅游消费，支持北川、安州、平武创建国家全域旅游示范区，支持仙海创建国家级旅游度假区，支持梓潼七曲山风景区创建国家5**旅游景区。持续开展绵阳名企名品省外“六进”活动，深度参与全省市场拓展“三大活动”。支持社会力量增加医疗、养老、教育、文化、体育等服务供给，更好满足人民群众不断升级的多样化消费需求。

加快建设质量强市。大力实施“质量强市”战略，力争成功创建“全国质量强市示范城市”，让“绵阳造”产品更受欢迎，让老百姓的生活更有质量。*开展质量提升行动，确保工业产品质量监督抽查合格率达94%以上，食品评价性抽检合格率达98%以上，主要农产品监测合格率、工程一次性验收合格率达100%。实施质量基础工程，力争主导或参与制修订国家和行业标准5项，新增省级企业技术中心3家。大力弘扬工匠精神，开展“绵州工匠”培育评选。实施品牌创建工程，积极争创中国驰名商标，新增商标注册1000件以上。加快创建省级农产品质量安全监管示范市。支持江油、三台申建地理标志产品保护示范区、盐亭申建农产品质量安全示范县、平武申建生态原产地产品保护示范区、北川申建有机产品认证示范区。

二、加快建设国家科技城

把握世界新一轮科技革命和产业变革大势，*实施创新驱动、军民融合发展战略，着力趟出一条军民融合路子，促进科技与经济深度融合。

保障服务重大国防科技项目建设。推动中国工程物理研究院某重大装置主体项目按期开工，支持中国空气动力研究与发展中心大型低速风洞加快建设、国家空气动力数值模拟军民融合重大工程项目年内落地开建，推动中国航发四川燃气涡轮研究院国家“两机”重大专项加快实施。支持九洲集团承担C919大飞机专项、国家高分专项等国家重大科技专项。务实推进与央属军工集团战略合作，争取更多国家重大科技专项和军民融合重大项目布局绵阳，力争再引进一批军民融合企业、院所和科研机构，进一步增强对经济建设和国防建设的整体支撑能力。

推进军民融合深度发展。完善军地院地联动合作机制，共同推动关键领域改革。打好全面创新改革试验收官之战，力争形成更多可复制、可推广的绵阳经验。支持在绵国防科研院所改革。积极推进国防科技重点实验室、军工试验设施、大型科研仪器向市场创新主体开放，打造共通共用、共建共享的军民融合示范平台。实施国家军民两用技术交易中心、大型仪器设备资源共享平台等市场化改造，力争完成技术合同成交额9.6亿元、平台整合仪器资源5000台（套）。积极争创国家军民融合创新示范区，争取国家首批军民科技协同创新平台布局绵阳。扎实开展科研人员激励计划试点。大力发展北斗卫星导航、核技术应用、新一代信息技术、航空航天、高端装备制造等军民融合产业。加快“军转民”“民参军”步伐，培育壮大长虹控股公司、九洲集团、攀长钢集团、东材科技等军民融合企业集团。持续开展军民融合企业认定，力争新认定军民融合企业50家以上、总数突破380家，军民融合企业产值突破1700亿元、增长12%以上。

建设国家创新型城市。围绕打造“双创”升级版，加快建设创新创业资源共享平台，拓展市场化专业化众创空间，力争科技企业孵化器面积达到115万平方米，入孵企业达到4000家。高质量建设长虹控股公司国家“双创”示范基地、西南科技大学“省部共建环境友好能源材料国家重点实验室”等国家级创新平台。*推进国家知识产权示范城市建设，新增专利申请量10280件以上、专利授权量4600件以上。继续实施高新技术企业倍增行动和科技型中小企业提质增效工程，新增高新技术企业50家以上，科技型中小企业稳定在1万家以上、新增升规入统60家以上。大力开展“创业绵阳”行动，办好第七届中国创新创业大赛军转民大赛等活动。加快建设智慧城市，让群众充分感受到科技创新带来的便利。

三、纵深推进改革开放

以改革开放40周年为重要契机，推动重点领域和关键环节改革取得新突破，不断提升对外开放层次水平，进一步增强发展动力活力。

*推进供给侧结构性改革。结合绵阳实际抓好“三去一降一补”，坚持用市场化法治化手段，严格执行环保、质量、安全等法规标准，化解过剩产能、淘汰落后产能。完善市场主体退出机制，妥善清理处置“僵尸企业”。积极开展“产融合作”国家试点。推进应收账款融资服务试点提速扩面，力争融资规模达到70亿元以上。继续落实减税降费措施，巩固扩大“营改增”政策性减税成果，推动水电油气运等行业要素价格市场化改革，让企业轻装上阵、聚力发展，为实体经济发展加油助力。

深化基础性关键领域改革。继续推进国有企业优化重组，稳妥推进混合所有制改革，完善现代企业制度，培育全国行业一流企业，推动国有资本做强做优做大。加快推动国资监管机构职能由管企业为主向管资本为主转变，有效防止国有资产流失。深化商业银行和农村信用社改革。争创军民融合金融综合改革创新试验区。统筹推进文化、教育、卫生等领域改革，充分**社会领域巨大发展潜力。

全面提升开放合作水平。坚持把招大引强作为投资促进工作的首要目标，突出先进制造业和现代服务业，大力开展精准专业化招商，力争引进30亿元以上重大项目9个、100亿元以上重大先进制造业项目3个，实现国内省外到位资金600亿元以上、继续保持全省第2位。构建符合当前发展需要的人才目标发展体系、人才政策体系、人才工作制度体系，办好第二届国防军工企业人力资源管理创新大会暨中国（绵阳）科技城军民融合人才峰会，运营好中国国际人才市场国家科技城（绵阳）分市场，力争引进培养高技能人才1.6万人，加快打造西部人才高地。建好对外开放合作平台，高水平办好第六届科博会等重大展会。加快推进中德产业创新合作平台建设。推进综合保税区申建，加快口岸物流通道建设。加强区域合作，主动融入“一带一路”、长江经济带，深化与京津冀、长三角、粤港澳大湾区等交流合作，积极承接先进制造业和加工贸易转移。主动对接省“四项重点工程”，参与成都平原经济区铁路公交化改造运营等项目，加快推进G5成绵高速扩容，推进安州区雎水铁路客、货站建设，推进南郊机场扩能升级。实施外贸优进优出“三大工程”，力争外贸进出口总额达到124亿元。

四、*推进新型城镇化建设

以人的城镇化为核心，以提高质量为关键，不断提升城镇综合承载能力和宜居水平，努力让居民生活更方便、更舒心。

建设现代化区域性中心城市。按照“建设新区、完善组团、提升旧城”思路，坚持规划、建设、管理并重，加快建设现代化区域性中心城市。积极推进绵阳城市总体规划修编。加快科技城集中发展区核心区“四横四纵”骨干道路和地下综合管廊建设，加快“科技之心”中央公园、市民中心、市文化艺术中心、核医学中心等公共服务配套项目前期工作，加快推进安昌河、草溪河综合治理。实施完善城市组团“五大专项行动”，完善菜市场、停车场、公厕等便民服务设施。启动实施三年棚改攻坚计划，开工城镇危旧房棚户区改造4275户、基本建成2939套。有序推进背街小巷整治和城中村、老旧小区改造，鼓励有条件的加装电梯。加快推进海绵城市建设。启动老城区部分行政事业单位搬迁计划。大力开展城市管理“五大整治行动”，确保顺利通过国家卫生城市复审验收。

支持县城和重点镇建设。*实施“宜居县城建设行动”，支持江油、三台加快建设现代化中等城市，支持梓潼、盐亭、平武、北川加快建设精品宜居县城，进一步增强县城综合承载力和辐射带动功能。深化拓展“百镇建设行动”，新创建省级试点镇7个。加快打造游仙区石马镇、梓潼县长卿镇等9个市级特色小镇。推进经济发达镇行政管理体制改革，培育县域范围内新的经济增长点。

加快农业转移人口市民化。落实户籍制度改革政策，促进有能力在城镇稳定就业和生活的农业转移人口进城落户，与城镇居民享有同等权利、履行同等义务。全面落实居住证制度，保障居住证持有人在居住地享有教育、就业、公共卫生等基本公共服务。建立农业转移人口市民化激励机制，切实维护进城落户农民在农村的合法权益。全年新增城镇人口5.6万人，常住人口城镇化率达到52.5%，户籍人口城镇化率达到35.5%。

全力推进基础设施建设。加快构建现代基础设施体系，为推进区域协调发展和新型城镇化提供有力支撑。持续推进交通攻坚，抓紧绵遂内铁路、绵苍高速、中遂高速前期工作，开工建设皂角铺铁路货场物流基地、S209改建等项目，加快建设九绵高速、广平高速、S205绕城改线、北川通航机场等项目，确保绵西高速、S416绵中路等项目建成通车。加快推进市县及县域之间快速通道建设。力争新建渡改公路桥8座、干线公路368公里。完成9个生活垃圾卫生填埋场技改扩容，开工建设绵阳市第二生活垃圾焚烧发电厂。确保乡镇污水处理设施实现全覆盖并逐步实现有效运行。加快推进武引二期灌区、开茂水库、沉水水库、“引通济安”引水工程等水利工程建设。继续推进骨干电网与智能配网协调建设和农村电网改造，完善天然气管网。推进通信基础设施建设，确保固定宽带接入用户总数达到148万户。

五、大力实施乡村振兴战略

坚持把实施乡村振兴战略作为“三农”工作总抓手，健全城乡融合发展体制机制，加快农业农村现代化步伐，让农业更强、农村更美、农民更富。

推进农业供给侧结构性改革。围绕粮油、畜禽水产等6个重点产业和生猪、麦冬等10个主导产品，建基地、创品牌、搞加工，力争新增现代特色效益农业标准化基地9万亩、现代农业产业园区12个。加快建设国家农业科技园，开展田园综合体建设试点。全面实施“沃野绵州”现代生态循环农业工程和优势特色农业提质增效工程，申报“三品一标”农产品100个以上。发展多种形式适度规模经营，力争新型农业经营主体达到1.75万个。大力推动农旅融合，建设一批设施完备、功能多样的休闲观光园区、森林康养基地、森林人家、乡村民宿。

全面深化农村改革。巩固和完善农村基本经营制度，落实好第二轮土地承包到期后再延长三十年的政策。全面完成农村各类产权确权登记颁证。落实农村承包地“三权分置”制度。探索宅基地所有权、资格权、使用权分置改革，适度放活宅基地和农民房屋使用权，提高农业生产要素配置效率。继续支持农村综合改革试验区建设。加快推进集体经营性资产股份合作制改革。推进农村普惠金融发展，加强支付惠农示范工程和农村信用体系建设，开展信用户、信用村、信用乡镇创建，推广供销社综合改革试点成果。落实藏粮于地、藏粮于技战略，全面强化粮食安全责任，扎实推进“两区”划定和建设。

推动农村各项事业全面发展。抓好“四好农村路”建设，新建改建农村公路1360公里。认真落实农村人居环境整治三年行动方案，加强农村突出环境问题综合治理，力争90%以上行政村生活垃圾得到治理。大力推广《农村居民建房设计图集》《农村居民建房技术导则》，新建幸福美丽新村300个、“四好村”300个。实施农村土坯房改造6万户，农村危房改造4400户。保护好具有历史文化价值的村落和传统建筑。完善乡村公共文化服务体系，加快建设基层综合性文化服务中心。侯卫东官场笔记

提升县域经济实力。更加注重分类指导，积极培育壮大县域特色优势产业，增强县域发展内生动力。支持县市区引进和实施重大产业项目，加快做大县域经济总量，力争县域地区生产总值达到1700亿元、占全市地区生产总值比重达到79%以上。出台促进民营经济加快发展的政策措施，加强产权保护，更好发挥企业家作用，让广大民营企业在市场经济的澎湃浪潮中大显身手。

六、着力推进绿色发展

坚持“绿水青山就是金山银山”，以对子孙后代高度负责的精神，加强生态文明建设，加快转变发展方式，加快建设天蓝、地绿、水清的美丽家园。

切实加强生态保护。严格落实主体功能区规划，统筹推进山水林田湖草系统治理，实施重要生态系统保护和修复工程。加强湿地保护，加大自然保护区综合整治力度，保护生物多样性。持续推进大规模绿化绵州行动，实施天然林资源保护和退耕还林工程，力争完成营造林50万亩，森林覆盖率达到54%。

注重资源节约利用。配合国家、省上划定并严守资源消耗上限、环境质量底线、生态保护红线，实施最严格的耕地保护制度，提高资源利用综合效益。大力发展绿色低碳循环经济，支持节能环保、清洁能源等产业发展。力争万元工业增加值用水量下降5.1%，单位地区生产总值碳排放下降3%。开展能效“领跑者”行动、节约型公共机构示范创建活动，大力倡导绿色低碳生产生活方式。

完善生态文明体制机制。制定经济发展环境保护负面清单和分区环境管控方案。严格环境准入，健全企业排污许可、环境信用评价、信息强制性披露等制度。加强生态环境监测网络建设，完善生态环境损害赔偿制度，继续实施涪江流域水环境生态补偿和水质超标资金扣缴制度，坚决制止和惩处破坏生态环境行为。完善县市区党政主要领导自然资源资产和环境责任审计制度。完成环保机构垂直管理制度改革。

七、全力保障和改善民生

把增进人民福祉作为政府工作的不懈追求，多谋民生之利、多解民生之忧，在发展中补齐民生短板、促进社会公平正义，不断提升人民群众获得感、幸福感、安全感。

切实办好民生实事。大力实施十项民生工程，办好19件民生实事，确保一般公共预算支出中民生支出比重稳定在60%以上。坚持就业优先战略，完善就业援助机制，扎实做好高校毕业生、城镇失业人员、失地农民和退役军人等重点群体就业工作，确保城镇新增就业4.7万人，使更高质量、更加充分的就业成为绵阳发展的突出亮点。加快建立多主体供给、多渠道保障、租购并举的住房制度，让更多群众实现安居宜居。完善基本养老保险及医疗、失业、工伤、生育保险制度，继续实施全民参保计划。进一步兜好民生底线，适时提高最低生活保障标准和特困人员供养标准，落实社会救助和保障标准与物价上涨挂钩联动机制，让每一个身处困境者都能得到社会的关爱和温暖。

全面发展社会事业。积极推进第三期学前教育行动计划。开展校外培训机构专项治理，着力解决中小学生课外负担重等问题。继续推进“义务教育薄弱学校改造计划”，覆盖学校增加25所、惠及学生增加1.64万人。顺应高考制度改革趋势，巩固提升高中教育质量。创新职业教育发展，鼓励大企业参与举办高质量的职业教育，促进产教融合、校企合作。加强师资队伍和师德师风建设。认真落实全民健身战略，加快南河体育中心升级改造，新建全民健身路径、农民体育健身工程100处以上，打造城市社区15分钟健身圈。推动学校体育、竞技体育和社会体育协调发展。认真落实《“健康绵阳2030”规划纲要》，加强公共卫生和重大疾病防控，巩固公立医院改革成果，推进分级诊疗和家庭医生签约服务，加强基层卫生人才队伍建设，提升基层医疗服务能力，推进中医药传承创新，优化生育全程服务。加快构建覆盖城乡的养老服务体系。全面加强残疾预防和残疾人康复工作，持续深化“量体裁衣”式残疾人服务。

全力促进文化兴盛。大力繁荣文化事业，加快发展文化产业，努力建设文化强市。弘扬中华优秀传统文化，继承革命文化，发展社会主义先进文化，培育和践行社会主义核心价值观。办好“感恩奋进”绵阳市纪念“5·12”汶川特大地震十周年活动。加强思想道德建设和群众性精神文明创建。繁荣发展哲学社会科学，鼓励优秀文艺作品创作，加快创建国家版权示范城市。加强文物保护利用和文化遗产保护传承，延续城市文脉。实施文化惠民工程，为人民群众过上美好生活提供更加丰富的精神食粮。

努力提高社会治理水平。完善基层群众自治制度，加强社区治理。发挥好工会、共青团、妇联等群团组织作用。促进社会组织、专业社会工作、志愿服务健康发展。完善公共法律服务体系，扩大法律援助范围。*实施“七五”普法，落实普法责任制，深化法治示范创建。加强和改进信访工作，深化网格化管理服务，有效化解矛盾纠纷。规范网约车和互联网租赁自行车管理。强化社会治安防控和公共安全体系建设，依法开展扫黑除恶和禁毒专项斗争，严厉打击违法犯罪活动，建设更高水平的平安绵阳。推动安全生产领域改革发展，坚决防范遏制各类生产安全事故。加大食品和农产品“双安双创”力度，争创“全国食品安全示范城市”，守护好人民群众“舌尖上的安全”。做好地震、气象、地质等工作，提高防灾减灾救灾能力。全面贯彻党的民族宗教工作基本方针，促进民族团结、宗教和顺，支持北川创建全省民族团结进步示范县。加强国防动员和后备力量建设，支持军队改革，做好双拥共建、人民防空工作。

八、坚决打好三大攻坚战

全面建成小康社会已进入决胜阶段，必须抓重点、补短板、强弱项，全力打好防范化解重大风险、精准脱贫和污染防治三大攻坚战。

打好防范化解重大风险攻坚战。完善市县金融监管体系，强化金融机构防范风险主体责任。加强风险监测和预判，密切关注银行不良资产、民间融资理财等重点领域，强化金融风险源头管控。依法做好打击处置传销和非法集资、整治互联网金融风险、清理整顿交易场所等工作，妥善处置企业信贷违约事件。建立更加严格的分行业资产负债率警戒线，推进市场化法治化债转股，有效防范化解企业债务风险。规范政府举债行为，推动融资平台市场化转型，有效遏制隐性债务增量，积极稳妥化解债务存量。

打好精准脱贫攻坚战。坚持以“绣花”功夫精准扶贫精准脱贫，聚焦“两不愁、三保障”和“四个好”要求，*推进产业、教育、健康、生态扶贫，补齐基础设施和公共服务短板，激发脱贫内生动力。确保北川贫困县摘帽顺利通过国家评估验收，高质量完成平武县摘帽、225个贫困村退出、35666名贫困人口脱贫的年度任务，巩固脱贫成果，让脱贫得到人民群众认可、经得起历史实践检验。全面推广“互联网+精准脱贫代理记账”模式。认真做好红原、壤塘藏区对口援建工作，扎实开展昭觉、布拖彝区扶贫协作工作。打好污染防治攻坚战。做好第二次全国污染源普查工作。实施打赢蓝天保卫战三年作战计划，开展大气达标攻坚行动，力争PM2.5年均浓度下降5%以上、全年空气质量优良天数达标率83%，努力消除人民群众“心肺之患”。落实“河长制”“湖长制”要求，大力实施良好水体保护等“四大工程”，国控、省控水质考核断面优良水体比例保持100%。加大土壤污染治理、修复与管控力度，实施好安州、江油土壤污染治理与修复示范项目。严格环境执法，强力整治群众反映突出的环境问题，守护好绵阳的绿水青山。

各位代表！

进入新时代，政府工作要有新气象新作为。我们将树牢“四个意识”，坚定“四个自信”，坚定维护以习近平同志为核心的党中央权威和集中统一领导，落实全面从严治党要求，加强政府自身建设，全面提升履职水平，努力建设新时代人民满意政府。

严格依法行政。推进依法施政，把政府工作全面纳入法治轨道。认真执行市人大及其常委会的决议决定，落实政府重大决策出台前向人大报告制度，自觉接受市人大及其常委会的法律监督和工作监督，自觉接受市政协民主监督，主动接受群众和舆论监督，推进审计监督全覆盖。健全行政决策机制，完善重大事项公众参与、专家论证等制度，加大政府信息公开力度，不断提高民主决策、科学决策、依法决策水平。

提升行政效能。按照中央和省上安排部署，稳步推进机构改革，加快形成职责明确、依法行政的政府治理体系。*落实《建设服务型政府行动方案》。深化“放管服”改革，全面实施“双随机、一公开”监管。推动综合行政执法体制改革。全面推行“互联网+政务服务”。加强政府诚信建设，依法兑现政府承诺。继续推进历史遗留问题化解，不断优化发展环境。

持续正风肃纪。推进“两学一做”学习教育常态化制度化，认真开展“不忘初心、牢记使命”主题教育。严格执行中央、省、市转变作风规定及实施细则要求，驰而不息整治“四风”，确保“三公”经费只减不增。推动政府系统党风廉政建设向纵深发展，严肃查处群众身边的不正之风和腐败问题，巩固反腐败斗争压倒性态势。进一步完善和落实正向激励和容错纠错机制，旗帜鲜明给积极干事者撑腰鼓劲，对庸政懒政者严肃问责。政府工作人员要履职尽责、勇于担当，清清白白做人、干干净净做事，决不辜负人民的信任和重托。

各位代表！新时代承载伟大梦想，新征程呼唤奋发有为。让我们更加紧密地团结在以习近平同志为核心的党中央周围，在省委省政府和市委坚强领导下，在市人大和市政协监督支持下，紧紧依靠全市各族人民，不忘初心、牢记使命，以永不懈怠的精神状态和一往无前的奋斗姿态，为加快建设国家科技城和西部经济强市，实现人民对美好生活的向往不懈奋斗！
